# An Account of Epidemic Erysipelas, as It Occurred at Columbia, Tenn.

**Published:** 1845-10

**Authors:** H. R. Robards

**Affiliations:** Columbia, Tenn.


Art. II.—*An Account of Epidemic Erysipelas, as it occurred at Columbia, Tennessee.* Read to the Medical Society of Tennessee, at its meeting in Nashville, May, 1845. By H. R. Robards, M.D., of Columbia, Tenn.

In lieu of the case which I was appointed to report at this meeting of the Society, I beg leave to submit a few remarks on the nature and treatment of a malignant and fatal epidemic which visited Columbia and its vicinity during the early part of the spring of 1844. So many accurate accounts of this disease as it occurred in various parts of the United States have been given by different writers, that it is with some degree of distrust that I venture to add to the number of reports and essays on the subject, nor should I have done so but for some peculiarities connected with its history as it prevailed in my neighborhood, which I have not seen mentioned by other writers. The facts connected with what I shall say of the disease are taken from notes made at the time, and very soon afterwards.

The first well-marked case that fell under my observation appeared about the last days of February. The weather had for sometime been dry and cold; the winter, with the exception of an epidemic measles that prevailed in the early part of it, had been unusually healthy, and there was not a case of this disease, so far as I am able to learn, within two hundred miles of that place in a straight line, at the time. This is one of the peculiarities to which I wish to call the attention of the Society, connected as it is with the causes of epidemics, a subject to which we cannot give too much attention. Why an epidemic erysipelas should, in its progress, have leaped over so many other places, and fixed itself upon a spot usually as healthy as those which escaped, is a question of great moment to every one, and of deep interest to the enquiring mind; but as I have no settled opinion to offer as to the causes of epidemics, I shall pass this part of the subject over, merely remarking that the notion of Sydenham, usually regarded as visionary, that the causes of epidemics have their origin in the bowels of the earth, is to my view nearer the truth than any one subsequently suggested. When a disease progresses in a given direction, or confines itself to particular localities or seasons, the existing causes of them may at least be inferred. The cholera, for instance, though exceedingly excentric in its movement, was thought to be most partial to such localities and seasons as afforded exciting causes which co-operate with the remote cause in the production of the disease. But in the case now under consideration that there should have been anything in the location of Columbia or in the weather, calculated to predispose or excite erysipelatous fever, can be determined only from the effects.

The symptoms of this disease, as it occurred in Columbia and its vicinity, correspond so accurately with those given of it by other physicians, as it prevailed in different parts of the United States, that I deem it altogether unnecessary to give at this time more than a few of the peculiar features by which it was characterized.

It was not only the fact in Columbia, but so far as I can learn wherever the disease has appeared, that the first cases were the most violent, and taking them as a focus the malady radiated from that point, and became less violent as it spread abroad.

The disease was almost uniformly ushered in by a chill, not very severe but protracted, continuing with alternate flashes of heat, in some instances, two or three days; this was usually preceded or accompanied by soreness of the throat. Reaction, when thoroughly established, was never very violent, and was followed by a protracted sweating stage; this generally brought the patient to about the fourth or fifth day, and during the time the tongue was covered with a whitish moist coat, and the pulse varied but little from the natural standard, of course slightly modified by the different stages. At this time, in a number of cases, the patient would seem to be rapidly convalescing, being able to get out of bed; he would now feel some thirst, or returning appetite; but if allowed to take a glass of cold water, or any mild article of food, from that moment there was no cessation of his sufferings, nor was any part exempt from the excruciating pains with which he was attacked. The peculiar nature of these pains I am unable to determine; in some instances they appeared to be neuralgic, as there was no external signs of inflammation; in others they seemed rheumatic, attacking the fibrous and sero-fibrous tissues; but there was no part exempt from one or the other, and when confined to the abdominal region were almost uniformly fatal, more particularly so when the uterus was attacked, which was generally manifested by menorrhagia; the discharge, however quickly subsided but the pain continued. The appearance of this discharge I regarded as a fatal symptom, and will venture to assert that it did not occur in a single instance which did not terminate in death. These pains often attacked the extremities, and so violent were they that the wretched sufferer would beg to have them severed from the body, if it would afford one moment's relief; after hours of intense agony nature would seem to become exhausted, and succumb to the very violence of the suffering. About the seat of pain in such cases there were often found circumscribed echymosed spots, and sometimes pendulous bags of effused grumous bloody matter. The form of the disease which I have been describing was very rarely attended with any signs of inflammation; the pulse was not inordinately excited, and, indeed, often did not vary from the natural standard; but the white coat on the tongue generally changed its color, and assumed first a yellow hue, and then became brown and dry.

This disease assumed another form which I will now describe: about the time I have mentioned, when a crisis seemed to be taking place, the inflammation which had been more or less violent in the throat, would make its appearance on some part of the face or side of the head; the alæ of the nose, the external ear, or the skin immediately over the parotid gland, were usually the first parts on which it showed itself; from a small circumscribed spot, unless arrested, it gradually extended itself over the side of the face and head, and finally attacked the opposite side. But this form of the disease has been so frequently described, and the external inflammation was so evidently a translation or a continuation of that from the fauces where it first occurred, that I shall not dwell upon it, but simply remark that the throat, though most frequently the first part attacked, was by no means uniformly so. The extremities were sometimes the first parts upon which the inflammation made its appearance.

As to the pathology of the disease, I have but little to say, but am satisfied of one fact, that erysipelas did often attack the mucous membranes, and extended itself either upon those membranes, or to the skin and the subjacent tissues.

Two important questions now present themselves for consideration:

1st. Was the disease propagated by contagion?

2d. What was the most successful practice?

As has been the case with almost every disease, now acknowledged to be contagious, there were many who denied its contagiousness; others who affirmed it. I believe, however, that there is no physician in Columbia, at this time, who does not admit that this disease, under certain circumstances, was contagious.

To prove this fact, I will take the first case that occurred as there was then no suspicion of a dangerous or contagious disease being amongst us, and as it was of long standing, many persons were exposed, more particularly as the weather was cold and the room consequently kept very close. Now, out of the number of persons that waited on that case, at least eight-tenths took the disease, two-thirds of whom died. But suspicions of its contagious nature having now got abroad, additional precautions were used, and in no subsequent case did so many take it from one patient; and, indeed, as I have before remarked, the power, of propagating itself seemed to diminish somewhat in proportion as it was multiplied.

That there were causes prevailing at the time, capable under favorable circumstances of producing the disease, is evident from many well marked cases having appeared where there was no exposure; still the risk of taking it was greatly increased by exposure, as was proved by the fact that when it attacked a family it was apt to go through it, and that several persons from an adjoining county, who visited their friends with it, took it themselves after they went home, and communicated it to others.

Now, the next question directly connected with the above is, could the disease be communicated in any other way than by exposure to the infected?

It would seem that this might be answered in the affirmative from the following circumstance: no case of it was known in the country until one of the physicians of the town, who was almost constantly in the rooms of those who had the disease, was called in the country to a difficult case of labor. He wore a blanket coat, as I understand, and was sometime confined in the room with his patient, during which time he rested himself upon a lounge or settee, which was occupied immediately after his leaving it by an old lady who was in attendance, who took the disease soon after and communicated it to some half dozen other old ladies, who visited her, every one of whom died. From these many others contracted it, and it became general and fatal in that neighborhood; indeed it was but little less so than in town, for during the space of five weeks, and in about five miles in diameter, about thirty persons died of it; but additional precautions being now used to guard against the contagion, the number of cases gradually diminished, and by the middle of May only its vestiges remained. The precautions that were used consisted in suffering as few patients as possible to occupy one room, and keeping that perfectly ventilated. The number of attendants afforded but little inconvenience, as the well were never over solicitous to be very near the patient. I am of the opinion that the disease was not contagious, except in cases of strong predisposition, or when there was long exposure in a close room. Those persons most obnoxious to it were evidently old women, and there were probably a larger number of females generally who took the disease than males, which may be accounted for by their being more exposed and going into the open air less frequently. Children under five years of age were almost entirely exempt from it.

The sequelæ of this disease may be worthy of notice. During the month of May there were only a few cases of it occurring, and by the last it had entirely disappeared in its genuine form; but then came on the summer fevers, different from the usual form and evidently complicated with the symptoms of the epidemic, but rarely to such an extent as to exhibit external erysipelatous inflammation, though there were many anomalous symptoms that rendered the cases very difficult to treat, and much more protracted than usual, many of them persisting four or five weeks. As the fall and winter came on the epidemic peculiarity became more manifest; sore throat was common, as well as fevers accompanied with bronchial inflammation and neuralgic pains in different parts of the body that were obstinate, and seemed rather to wear themselves out than yield to any treatment.

I come now to the most important point connected with all diseases—its treatment. The disease which I have been describing is evidently of a specific character, and hence many have supposed that it requires a particular system of practice, and that no other will be successful, whether in one season or another, and under all circumstances. The fallacy of such opinions will appear evident from the success attending the treatment of this, as adopted by different practitioners in different seasons and different localities.

One physician, who lives in the north, will tell us that he treats the disease successfully with the lancet, emetics, and purgatives; another, being in a different latitude altogether, and in a different season, adopts the same plan and pertinaciously adheres to it. Every sensible practitioner, it would seem to me, must at once perceive how dangerous it is for us living in this latitude to be governed by the experience of any man living in one five, six, or eight degrees farther north or south, in the treatment of any epidemic. And until constitutions, temperaments, habits, and localities shall cease to exercise any influence over diseases, and until a specific mode of treatment shall be defined for each particular disease according to its name or class, until each constitution will bear the same amount of depletion and the same extent of stimulation, in vain may we expect the same results from any system of practice in the north and in the south, and in all the seasons of the year; and as no routinist was ever a successful practitioner, so neither can the copyist ever be. I have heard it asserted, by a distinguished physician, that the Americans were the most successful practitioners of medicine in the world. If this be true, it may be attributed to the fact, that they, less than any other nation of people, adhere to the tenets of any particular doctrine, judge more for themselves, and treat diseases according to their indications, regardless of the theory of any man or set of men. It is to be hoped that the physicians of the south-western country are beginning to investigate diseases for themselves, and trust less to the teachings of the schools. We are told by one or two writers in the northern and eastern parts of the United States, that this epidemic was treated there most successfully upon the strict antiphlogistic plan. By others, living in different sections, we are assured that the mild and expectant plan in the early stage, and tonics in the advanced stage, was the only successful one. Now, these contradictory statements will seem much less preposterous if we will take into consideration the modifying influences of climate, locality, season and population. That the disease now under consideration, or any other of specific character, should assume such a variety of forms and types, as to demand an opposite plan of treatment as it occurs in different localities, seasons, and climates every one will admit who has observed at all, or at least who has paid the slightest attention to this important subject. No one in his right senses would think of treating a case of malignant intermittent fever in this latitude, in the same manner as experience has taught the southern physician it must be treated, in the southern part of the United States. Here, as my own experience has proved to me, we may bleed not only with safety, but in many instances with the effect of cutting short the chill at its very accession.

Such practice, there is good reason to believe, would be most inevitably fatal farther south. Again, we are told by Dr. Graves, that erysipelas prevailed in Dublin as an epidemic one season, and demanded the most active antiphlogistic treatment, but that as it prevailed the next season, tonics and stimulants alone could be relied upon from the very commencement. I might refer to many examples of a similar nature, and to a thousand instances of the fatal effects of persisting in a system of practice that has proved successful in one locality or season, and therefrom arguing that it must do so always and every where. What, for instance, has gone with Dr. Rush's far famed "ten and ten," in the treatment of yellow fever? Why is not the lancet in the hands of every practitioner in the south, as directed by him and Dr. Smith of London, in the treatment of fever? Why is not the cold bath uniformly successful in the treatment of typhus fever, as it was in the hands of Dr. Currie? Why has it not been so even in Europe since the one or two seasons it was so extensively used there? Why, in the treatment of congestive fever, in this climate, do we not go armed with a phial of quinine in one hand, and of capsicum in the other, and bid defiance to its terrors, as the southern practitioner does? I need not tell those who have tried it, that it will not do. Again, why do we not fear, in the treatment of all fevers in this country, at this time, the effects of emetics, saline purgatives, watery discharges, collapses, &c., as we did so justly two or three years after the cholera had swept over our country, and left its remains to modify every disease that occurred? I need not say to every practitioner present, that the causes which produced a tendency, in almost every case that occurred of whatever nature, to those gastro-enteric affections has gradually but perceptibly disappeared, leaving behind no other evidence of their having existed, stronger than a groundless prejudice against remedies which, under different circumstances, are most useful. I have dwelt upon these preliminaries before proceeding directly to the treatment of this disease, because I deem them of importance, and I am confidently of the opinion that a want of attention to these facts has done more to bring disrespect upon the profession, and subjected it to more slurs and opprobious epithets than any thing else. Why do doctors so often differ, and why is there so much uncertainty in the practice of physic? There are many diseases, of course, beyond the reach of our art, and many so obscure that a correct diagnosis is most difficult; but I believe if every physician when he sets out, had to study general principles alone, and afterwards learned at the bedside of his patients to discriminate and mark the changes that modifying influences are constantly producing in the character of diseases, much less difference of opinion would exist, and far better success would attend the practice. I presume there is no one who has treated as many as half a dozen different epidemics, without having felt what that most accurate of all observers in medicine, Sydenham, confessed he experienced on the first appearance of every epidemic, namely, a great difficulty in determining what course would be most successful, and the painful necessity of experimenting with the first four or five patients before he was satisfied. Now how much better to do that than to form an opinion at once and adhere to it throughout, whether right or wrong. I do not suppose there is a gentleman present, who does not know that the diseases of this country are constantly changing, and that in no two years do they require precisely the same treatment. How erroneous it is, then, to say that because a disease is inflammatory, we must bleed and purge actively; or that in fevers any one remedy, or any class of remedies, must invariably be used.

Measles is an inflammatory disease, for instance, and I have seen it bear, and even demand bloodletting and purgatives; yet as it occurred in Maury county, two winters ago, that practice was uniformly fatal; hence it became almost proverbial that old women treated the disease better than the doctors. The same thing I have known in scarlet fever; I have also known the subjects of the summer and autumnal fevers of that county to bear bloodletting well one season, and sink under them the next.

But I must now come to the treatment of erysipelatous fever. As may be inferred from the foregoing remarks there was a variety of opinions among the physicians of Columbia, alike as to the nature and treatment of this disease. Some regarded it as a purely inflammatory sore throat, and treated it by active depletion—bled, puked, purged in all instances; others regarded it as a modfication of vernal intermittent and treated it accordingly, keeping in view, however, the inflammatory character. One of the number thought it typhus, and gave tonics and stimulants after the first few days. But the state of things presented by our town so accurately corresponds with what Dr. Ramsey, a very intelligent physician of Knoxville, in answer to some questions I put to him relating to the treatment of the same disease as it appeared in that place, describes, that I will give it in his own words. "The treatment," he says, "was in accordance with the pathological views before mentioned. One physician bled, and purged with jalap; another bled, and, in some cases, he thought, with advantage, but in others with manifest disadvantage, as great depression followed even the loss of a small quantity of blood, while another prescribed with a care, almost approaching to the expectant practice. The comparative mortality from the first two methods of practice I cannot give, for I do not know the mortality of the disease as shown by the symptoms of the patients thus treated; but more patients died who were treated on the vigorous antiphlogistic plan, than of those who were treated by the plan indicated by rational observation. A comparison cannot be drawn between the expectant practitioner and the others, for he had not as many patients under his care; but he did not lose a patient. He used laxatives after one purge, and very small doses of ipecacuan as a relaxant, not in sufficient doses to nauseate, and upon the subsidence of the febrile and inflammatory symptoms, gave stimulants."

Such I have said was very nearly the state of things in Columbia. My own observation and experience sustain the observation of Dr. Ramsey, that more patients died who were treated on the vigorous antiphlogistic plan, than of those who were confided more to the powers of nature.

It will be remembered that I have noticed two forms of this disease;—one in which the inflammation made its appearance externally; and another which was attended with violent neuralgic or rheumatic pains. As to the latter, I do not know what would have been the best practice, for every case that I saw or heard of, in which those pains were violent terminated fatally. I saw but one, and the subject of that was a young lady. I treated the case at first with a brisk purgative, and afterwards with broken doses of calomel and ipecacuan, anodynes, and the warm bath. The disease attacked the stomach and bowels in the first instance, and afterwards extended to the uterus. It was after the fourth or fifth day of the disease that her sufferings commenced, but from that time to her death, which occurred on the tenth, the only thing that gave her one moment's relief was the warm bath; and that was only temporary. Anodynes made no impression. I will mention one peculiarity about the tongue in this case. At first it was lightly covered with a yellow bilious coat; but about the same time that the pains in the abdomen commenced, the tip assumed very much the appearance of plum jelly; the redness then extended along the edges and up the middle. Her grand mother, an old lady, had the same form of the disease; though in her case the pains attacked the extremities; she was bled and purged freely, but lived only a few days. There were others who were treated very actively, and in all the result was unfavorable. As to that form of the disease which attacked the throat, and extended to the face, head, &c., and which was by far the most common, my experience was that it was easily treated, since I did not lose a case of that kind.

My practice consisted chiefly in relieving the biliary symptoms, which were constant, by a brisk calomel purge, followed by a dose of senna and salts, or cream of tartar. Afterwards my aim was to keep up secretory action. I usually gave very small doses of calomel and ipecacuan, and occasionally a small quantity of senna tea, to carry off the accumulation of vitiated secretions from the bowels. This course was often varied according to the indications, but in no case did I bleed profusely, and but once at all. Emetics may have been of service, but I never used them. For the throat I sometimes used the chloride of lime, and at others, tincture of myrrh and creosote. To arrest the inflammation in the skin I sometimes surrounded it with a strip of blistering plaster, but believe that it was best to cover the whole inflamed surface with a blister, for as soon as the suppuration was established the disease generally subsided.

It may be asked why I did not bleed and purge more freely? My reply is, because the indications were not that way. The excitement of the pulse was not commensurate with the other signs of inflammation; and, besides, I saw at an early period, that those who bled were not apt to cure their patients.

Columbia, Tenn., 1845.

